# The Atlantic Areas and the Eastern Mississippi Area

**Published:** 1917-03

**Authors:** Felix Von Oefele

**Affiliations:** New York


### The Atlantic Areas and the Eastern Mississippi Area.

DR. FELIX VON OEFELE, New York.

The central ridge of the four areas mentioned is a mountain chain of crystalline rocks since palaeozoic era. It is somewhat irregular in the northern as well as in the southern part. The declines of the ridge are ribbons of younger deposits.

The Canadian Shield of azoic crystalline rocks and the eastern part of the central ridge mentioned contain rocks exposed for long periods to atmospheric waters of the surface and strongly extracted; or where the appearance on the surface of earth is due to glacial influence, it was exposed to many extractions previous. The eastern mountain decline descends quickly to the seashore. It is exhausted by the circulating water of all the geological periods. The conditions were always unfavorable for large deposits of rock salt or strata of other easily soluble material. For this reason, the springs of the three Atlantic areas are commonly low mineralized. Charleston Artesian Well, South Carolina, as a tepid sodium chloride water of second area and Lubee Spring, Maine, as a brine in the third area are exceptions.

The volcanic action has stopped since the middle of mesozoic time, in the areas mentioned.

The mesozoic volcanic action made intrusions through the three Atlantic areas. The respective districts yield many waters claimed as mineral medicinal waters and bottled as table waters. But no water of these districts is tepid or really thermal. This seems astonishing.

The older precambric volcanic action was much stronger. Its intrusions are much larger and its districts still yield few springs of higher temperature. The tepid temperature of the springs of Tahonic Mountains of the third area and the tepid carbonated springs of Charleston, South Carolina, mentioned appear in younger rocks, but seem to originate from covered precambric intrusions. The Eastern Mississippi area shows many springs of higher temperature originating directly from these precambric intrusions.

The vicinity of New York city shows specimens of three kinds of intrusions. The serpentine hills of Staten Island correspond to the older precambric intrusions. The Watchung Mountains of New Jersey contain mesozoic intrusions. The Palisades are of intermediary age.

During the geological periods, the northern parts of the Atlantic areas show the ocean progressing into the continent. There the kaenozoic, mesozoic and younger palaeozoic strata are lost. This is the New England area. There the oceanic decline is built by rocks of old geological age. It is not always clear which of these rock springs are stratum springs and which are fault springs. We will speak of the silicated springs as stratum springs and of the nitrated ones as fault springs. As a rule all the waters of the New England area are strongly submineralized. Mineral springs of the two other Atlantic areas are somewhat higher mineralized.

The entire waters of the combined Atlantic areas are ordinarily below the average of total mineralization of good drinking waters of European standard. There is no occurrence of calcium sulfate springs, sodium chloride springs with the two mentioned exceptions nor especially of sulphide springs which are very frequent in the Eastern Mississippi area including the upper part of New York State.

The Atlantic areas had emerged in a long geological period from sea bottom, and part of them at different times again submerged and reemerged. The combined Adirondack and Appalachian system of palaeozoic time was a broad island of very high mountains in the north and became narrow in the south. The marine deposits of strata east of this island form the three Atlantic areas.

I. THE MIDDLE ATLANTIC AREA.

In the Middle Atlantic Area, the palaeozoic Appalachian island has had the seashore to the east may be within the eastern plains of the present continent or further to the east in the ocean of today. In the proterozoic period there was a strong volcanic action in New Jersey and neighborhood up to New England and down to the southern Appalachians. The seashore of the geological Appalachian island supposed, in the west gradually spread in the direction to the Mississippi. In the east the island submerged by and by to such a degree that at the end of the palaeozoic preiod almost the entire State of New Jersey with the exception of a small district was under water. With the beginning of the middle mesozoic period, a new area of volcanic action took place and gradually the eastern seashore had emerged to the extent we find it now. After the middle part of mesozoic time, the volcanic action stopped forever in the east. A similar development, but less regular happened north and south of New Jersey with the above mentioned modification.

The Middle Atlantic area includes Long Island, Manhattan and Staten Island, N. Y., New Jersey, Delaware, a small eastern half of Maryland, District of Columbia, the plains of Virginia and the northern plains of North Carolina.

The Middle Atlantic area must be subdivided into 1) the Pleistokaenic district of marine deposits, 2) the Meiokaenic and moraines district, 3) the isolated Oligokaenic springs of Long Island, 4) the Cretaceous district, 5) the old mesozoic district and 6) the Palaeozoic district.

The end of the palaeozoic period may be called the centurial high tide, the water covering nearly the entire State of New Jersey, while with the beginning of the mesozoic period a new low tide started.

If we include with the State of New Jersey a small southeastern part of the State of Pennsylvania, there is the most regular deposit from azoic to recent periods represented from the summit of the Appalachian system to the seashore. New England shows in the north only the older kind of palaeozoic deposits and the states south of Carolina show only the more recent kaenozoic deposits.

The districts of the Middle Atlantic area are almost regular strips of all geological ages. They show stratified marine deposits on the front of the old rocks with an original decline like the Andes to the Pacific Ocean. Three old periods of parallel volcanic intrusions and a following gigantic outlet of the prehistoric Hudson glacier, a spur of the inland ice cover, separated the Middle Atlantic area from New England. No thermal or tepid spring still exists from the intrusions of the Middle Atlantic area.

The State of New Jersey has only a total area of 7815 square miles. The northwestern part is furrowed here and there, by ridges of the eastern decline of the Appalachian Mountain system. The remainder is low situated land falling in an eastwardly direction toward the seashore. The southern half, particularly that portion south of Trenton contains only very low hills and even land. The same condition prevails in the remaining part of the Middle Atlantic area south of New Jersey. According to the surface strata, the entire State of New Jersey may be divided and subdivided into ribbon like strips, running northeast to southwest, of different geological character.

The geological map of the state geologist, Henry B. Kuemmel, and the topographer, C. C. Vermeule is edited under the direction of the Geological Survey of New Jersey. The palaeozoic district of New Jersey has an area of 1546 square miles, the triassic district an area of 1779, the cretaceous district an area of 970, the meiokaenic district an area of 3086, the pleistokaenic district an area of only 45 and the swamps and morasses between the meiokaenic and pleistokaenic district are extended over an area of 388 square miles. These different districts have very many subdivisions consisting of rocks and soils which vary considerably, giving to the waters which course through them, quite a range of properties. But the general character persists through the single districts.

But usually all the waters of these districts claimed as medicinal waters, are below the mineralization of European average drinking water. The field of meiokaenic marl deposits yields highly mineralized waters, but objectionable by reason of the dissolved constituents contained. These highly mineralized marl waters can not be claimed as medicinal or mineral waters. That is the reason, why we will learn of no mineral waters from the largest i.e. the meiokaenic district of State of New Jersey.

AREA I, District 1.

From Long Island, N. Y., to the northern plains of North Carolina, there is a continuous ribbon of kaenozoic deposits. Similar conditions prevail again in the southern states down to Florida. But the kaenozoic deposits are interrupted between North and South Carolina.

The pleistokaenic deposits consist of marine sands. Since these sand strata are exhausted by marine influences, their springs contain a very low amount of total solids and are very pure and clear. The small islands east of Long Island and the field of Cape Cod itself partly show this formation. But there is no claimed mineral spring.

A small ribbon of pleistokaenic sand extends from Sandy Hook, N. J., to Cape Hatteras, N. C. North the sea made no estuarine deposits or they are covered by glacial drifts. Only the New Jersey coast shows deposits of estuarine pleistokaenic sands. This is a field of desirable very low mineralized surface springs.

The pleistokaenic or quaternary marine deposits of New Jersey have a total area of 45 square miles. These deposits running parallel to the coast of New Jersey and comprising parts of the mainland, are approximately 120 miles long, varying in width, being 1 to 1½ miles at its widest part. This includes part of Monmouth, Ocean, Atlantic, and Cape May Counties from Sandy Hook in the extreme northeastern part of New Jersey to Cape May, the furthest southern point of the state.

The quaternary deposits composed chiefly of glacial deposits and waste which were brought down to the shores from the continent by the glaciers and rivers are not included in this district. We are concerned chiefly with the beach sand and gravel, washed up by the sea, including dunes and dune sand along the coast, which is not always sharply distinguished from Cape May formation.

This beach sand which is being continually thrown out by the ocean, is silicon dioxyde in a very pure state. Time and the sea water have had their effect upon the sand and the result is that the sand is free from almost all mineral salts except a small amount of arduously soluble lime salts which are there due to the presence of shells, etc. Some places are richer in lime salts, some almost free, depending upon the neighboring banks within the sea. The waters of the springs, as they bubble up through the sand, are mixed with air or aerated and as almost all salts in the sand are already gone they dissolve but a minute portion of mineral matter.

All the waters of this formation are similar to a certain extent. I took for examination a specimen of drinking water from a well at Trotters, near Brielle, New Jersey. The well is located in a deep stratum of pleistokaenic sand, the water being raised by a pump. Upon a preliminary analysis I found the following data: total solids 0.01085%, loss by ignition 0.00223% and Calcium oxyde 0.00223%. These are figures of akratopege.

Different mineral springs are alleged to be in this district of New Jersey and carrying on active business with the consuming public, i.e. the sale of bottled waters.

Upon reference to the 37th Annual Report of State Board of Health of State of New Jersey, 1913, pg. 448, table 79, there were three springs of the Pleistokaenic district selling bottled water, namely, Cold Indian Spring at Asbury Park, Indian Lady Hill Spring at Asbury Park and Indian Spring at Rockaway.

Indian Lady Hill Spring 0.0056% t. s.

Cold Indian Spring 0.0062% t. s.

Delaware contains the same geological condition as eastern New Jersey; but it does not claim to contain mineral springs. In 1911 one spring reported actual sale.

The State of Virginia is principally divided by meridian 77° 30′ into an eastern kaenozoic and a western proterozoic district of deposits. The kaenozoic part of Virginia contains very few claimed mineral springs.

North Carolina contains no claimed pleistokaenic mineral spring.

AREA I, District 2.

The waters of the Moraine deposits and of meiokaenic strata are more highly mineralized. The pleistokaenic deposit of Long Island borders on the upper Cretaceous deposit. Meiokaenic deposit is not known north of New Jersey and east of the eastern mountains. A broad strip of Meiokaenic deposit occupies quite an extensive district of 3087 square miles of the State of New Jersey. This strip is continuous to the south through the middle and southern Atlantic areas. The soil of this strip of New Jersey is covered chiefly by moraines and glacial drift and is quite rich in soluble minerals. Nevertheless it boasts of but two claimed mineral springs, the Cold Springs at Brown Mills and the Artesian Well at Winslow.

Of these which are called Meiokaenic deposits, there are various sedimentary Rocks, partly of marine origin, partly river deposits, partly glacial drift. Some may be really Pleiokaenic, some Meiokaenic, some really Eokaenic. The marl character or the marl admixture of these deposits spoils the properties of the water. The absence of acknowledged springs serves but to emphasize the fact that the Meiokaenic strip of New Jersey with its 3087 square miles has little or no mineral water of any therapeutic value.

Upon correspondence with the above, I received a reply from the Cold Springs, but none from the Artesian Well. Because of its high mineralization, the waters are not beneficial as a drinking water, for, instead of acting as a diluent or flusher, they do just the opposite. The Meiokaenic deposits of New Jersey give undesirable properties to the water. As for the Artesian Well, it is fair to presume that since I received no answer to my letter, and since I could find no evidence of there being any water by this name on the market, that the spring has not been in existence for some time. The Bulletin of New Jersey State Board of Health erred therefore in mentioning it as one whose waters are being sold on the market. This is sufficient evidence that this large Meiokaenic district does not contain a spring able to continue the claims of a mineral spring for even a decade.

Mardela Springs is a spring of Maryland Ocean plains of Meiokaenic district. Virginia is not yet clear. North Carolina contains the meiokaenic springs: Indian Spring, Albutton Mneral Spring and Cowhead Spring.

AREA I, District 3, oligokaen.

The conditions were the same in old periods northeast of New Jersey up to Cape Cod. But the eruptive time of the serpentine rocks of Staten Island, then of the Palisades, then of the Watchung Mountains, then of the Hudson glacier and the absence of recent estuarine dunes destroyed the similarity to the conditions of New Jersey.

Long Island with a few other small islands is the only residue corresponding to the stratified ribbons of New Jersey and more south. The northwestern quarter of Long Island is a cretaceous formation. The rest is pleistokaenic formation belonging to the second district. The principal part is a southern and a northern long row of end moraines of the pleistokaenic Hudson glacier. Where this glacial drift does not cover exceptionally older kaenozoic hills, they consist of a very pure quartz sand. The glacial drift does not contain waters desirable for medicinal use. The moraines yield average mineralized waters of undesirable chemical constitution belonging to the second district. The boundary of Nassau County and Suffolk County shows an uncovered ridge of oligokaenic sands with some Akratopeges.

Momone Spring and Colonial Spring at Wayandanch were used as medicinal springs by the old Indians of Algonquin tribes and until half a century ago then too by the settlers. Wrong cultivation increased the amount of mosquitoes and kept the visitors away. Oyster Bay Spring Water from the neighborhood of Oyster Bay is on the market as table water. These three are very low mineralized springs in Suffolk County, N. Y.

The waters of the moraine deposits are more highly mineralized.

A well of Ozone Park at Woodhaven, L. I., 0.0162% total solids.

A rejected plant well at Brooklyn Hills, L. I., 0.0436% total solids. Earthy salts prevailed in both samples. Both these wells are a few hundred yards distant and show the difference of soluble contents within the glacial moraine hills. They claim no medicinal spring.

AREA I, District 4.

A small ribbon of Cretaceous district is known in the northwestern part of Long Island. A more or less continuous strip of Cretaceous deposit extends from New Jersey to South Carolina.

The cretaceous district of New Jersey is a long narrow ribbon-like strip of land running in a northeasterly to southwesterly direction. The four corners are New Brunswick, Monmouth Beach, Kelley Point and Salem. From Trenton to southwest, the boundary line is the Delaware River running there in a geological fault. This cretaceous district of New Jersey contains 970 square miles or about 1/8 of the total state. This cretaceous district is fairly well populated. It includes Camden, the main part of Trenton and New Brunswick.

In the records of Washington and New Jersey and in other publications, at different times we find the names of springs which purport to be good for medicinal and drinking purposes. It was possible to collect the following springs of the Cretaceous district of New Jersey, which have at one time or another been claimed to be mineral springs: Arctic Spring Water at Trenton Junction, Gray Rock Well at Ewingville, J. S. Hart Natural Spring at Lawrenceville, Sanhican Spring at Trenton Junction, Wm. S. Potter Well at Hamilton Township, Salem-Art-dist-pure Water of H. A. Frye Co. at Gloucester and Kalium Spring Water at Collingwood.

I have tried to obtain information from the proprietors of these springs regarding their qualities, etc. A letter addressed to the Crystal Spring Water Co. was returned as unknown, and Potters Well was unheard of by the New Brunswick Board of Trade. The Arctic Spring Water could not be located. Satisfactory answers were received from J. S. Hart, A. B. Vernam and Wm. Lawver. Not one of these springs could supply me a chemical analysis of their water and from the Sanhican Spring only I received a copy of a very incomplete sanitary analysis. Chlorine was stated present 0.0004%, Nitrogen as Ammonia 0.0000018%, Nitrogen as Nitrates 0.0002% and Oxygen is consumed by permanganate in solution 0.0000054%.

Upon special request I obtained samples of water and made such tests as would enable me to classify these waters in a general sense. The Sanhican Spring showed: 0.0082% total solids, 0.0069% mineral matter, 0.0013% disappeared in red heat and 0.0041% Calcium Oxyde. Magnesium Oxyde was found present in traces.

An analysis of a sample of Echo Spring Water Co. at Ewingville shows 9.0084% total solids, .80024% mineral matter, 0.0080% disappeared on heating and 0.0012% Calcium Oxyde. It can be learned that this spring yields a relatively higher amount of organic matter. But the ash of Sanhican Spring is reddish colored by iron and the ash of Echo Spring shows no coloring by iron and therefore is more valuable for many technical purposes.

The water of J. Hart Spring showed 0.0093% total solids and 0.0021% calcium oxyde.

In comparing the analyses of these springs we find their mineralization very low, if we consider that a good European average drinking water contains between 0.04 to 0.05% total solids. The above springs of the cretaceous district of New Jersey contain almost 1/5 only of this average, and must be called submineralized waters of a very low mineralization. The cretaceous district offers no wonderful possibilities of spring development, but contains fairly good waters for ordinary use. But it is of vast importance to develop similar springs and mineral waters, owing to the close proximity of said district to the city of New York, Philadelphia and other large cities and suburban centres in the vicinity.

The geological conditions of this district are not suitable for high grade medicinal mineral waters; in other words they may be correctly termed only relative mineral waters. The soil contains remarkable quantities of calcium compounds, mixed with glauconite sand and iron oxyde. Marl is also found in traces; this marl imparts a bad taste to the water.

Wm. J. Lawyer of Trenton Junction has given me valuable information covering the springs of this section. He says that those springs located inland from 1/4 to 3/8 mile of the Delaware River are clear while those further back from the river are both poorer in quality and quantity.

This is accounted for by the water coming in contact with a stratum of soft red shell composition which shows in the analysis of the water to its disadvantage. For a stretch of four miles on the Jersey side of Delaware River the quality of the water is excellent, the land being of a rich clay loam composition intermingled with a small percentage of large grains of sand. The clay is white and pliable. The bed rock is either brown or gray stone of a very solid formation suitable for quarrying. Thus the water is cleansed of all impurities through a natural filtering process brought about when the water comes in contact with the sand and clay.

Bath's Chalybeate Spring in Bucks County, Pennsylvania, is situated in the fault between Precambrian and Lower Cretaceous. A very small southeastern part of Pennsylvania near Philadelphia contains Precambrian crystaline rocks, Cambrian deposits and Precambrian intrusions as island within Cretaceous deposits. No mineral springs are claimed there.

Baltimore and Washington show cretaceous deposits and precambric intrusive rocks. Springs near Baltimore are: Strontian Spring, Indian Spring at Hillsdale, Magnetic Strontian Springs, Clymerara Springs, slightly distant; Bentley Springs and Windsor Sulphur Springs.

The District of Columbia contains: Berry Hill Mineral Springs, Columbia Natural Lithia Springs and Gitchie Crystal Spring (0.0022% t. s.)

Bladsburg Spa, Spa Springs, Takoma Spring and Rockville Spring are near Washington, within Maryland.

Perhaps Chase City Lithia Spring and Buffalo Springs, Mecklenburg County, belong there. This would depend upon the neighboring springs of North Carolina.

Virginia contains two small separated fields of cretaceous and triassic deposits and no palaeozoic deposits at all on the eastern decline of azoic rocks.

AREA I, District 3.

With the beginning of the middle mesozoic period called the jurassic period the last volcanic action took place in the east of the United States and by and by the eastern seashore of New Jersey, etc., emerged in the periods explained above to the extent at which we now find it. The jurassic volcanoes were all located near the seashore of their times. The respective intrusions are proved in fields of old mesozoic deposits called triassic deposits. After the middle part of the mesozoic time, the eastern volcanic action stopped forever.

The end of the palaeozoic period may be called the centurial high tide, the water covering nearly the entire state of New Jersey and similar country, while with the beginning of the mesozoic period, new low tide started. The Cretaceous district and the above mentioned more recent districts contain strata which were deposited after volcanic action had ceased. In these districts mineral springs ascending in an old volcanic fault do not reach the surface of the earth save in exceptional cases. We learned above the exception of Charleston Artesian Well, South Carolina, as a tepid sodium chloride water of the second area reached by thrilling. The waters of later geological deposits contain on this account very little or no radioactivity. But the waters of triassic and older deposits may contain an admixture of magmatic waters and radioactivity.

The triassic deposit of New Jersey is a strip with many volcanic intrusions. A higher radioactivity is often the property of the springs of such districts. But the radioactivity is still of small importance for practical balneology. It is almost a matter for bluffing advertisers. The amount of trustworthy statements of radioactivity are insufficient. The following pages will mention the radioactive properties only exceptionally.

Taking into consideration all the spring groups of northeastern United States, we can expect in the triassic district of the first area only low mineralized springs, or springs influenced by the old volcanic action. This volcanic action has lost locally every influence to the temperature of mineral spring waters, so that no high mineralization of magmatic waters can come to the spring in existence. This mesozoic volcanic action of New Jersey is far distant from the precambric center of the Adirondack system as well as from the central point of the Appalachian system in Virginia. Within New Jersey the volcanic influence may cause a very high dissociation or some radioactivity. As far as low mineralized springs are concerned, the best are to be expected in the triassic district of New Jersey.

The small ribbon of triassic deposit known in Massachusetts and Connecticut in a north to south direction and the other end down to Virginia are accompanied also by volcanic intrusions. The New Jersey triassic district overleaps into the state of New York. Cascadian Spring of Grand View, Rockland County, New York, may belong to the same group of waters of the triassic district of New Jersey.

Looking through the annual report of the Board of Health of New Jersey for the year 1913, I found that mineral waters of twenty-two springs of the triassic district were on the market for sale. On looking through other publications I could not find any additional springs than those tabulated in the Board of Health report.

The springs, of the axis of this ribbon marked from Lambertville to Gettysburg, in the vicinity of Philadelphia are partly mesozoic. Its next ribbon with bottled waters is triassic deposit with mesozoic intrusions similar to Watchung Mountains in New Jersey. These springs are Pavillion Spring, Bercks County, Ephrata Spring, Glacier Spring and Black Barren Spring in Lancaster County, York Spring and Gettysburg Katalysine Spring in Adams County, Pennsylvania.

Virginia shows a small isolated field of the same conditions in Fauquier and Culpeper County with three claimed mineral springs.

North Carolina, South Carolina and Georgia contain almost no triassic deposits. There was a large district of precambric crystalline rocks situated near the seashore in mesozoic time and this district contains the same mesozoic intrusions as the triassic district of the Middle Atlantic area.

I wrote to all the twenty-two springs of the district of New Jersey. Five have answered, sending me reports of the analyses of their water. One other answered a long time ago. A few of the remaining springs could not be located by the Postmaster; they were four in number, and the letters were returned to me as unclaimed. The rest did not answer at all. A few of the remaining twelve springs do not seem to live up to the requirements of natural medicinal spring water. The majority of these waters seem to be trustworthy and reliable. Evidently the owners do not understand the high value of the publicity of their analyses.

The analyses received from the above mentioned six springs are recalculated to the decimal system and free ions and they are tabulated below. Alco Spring, Watchung Spring, Rock Spring, Culm Rock Spring belong to regular field. Trinity Spring is in relation to the Palisades intrusion and the Englewood Spring is influenced by glacial drift.

The volcanic triassic district of New Jersey furnishes waters of a homogeneous character of very low mineralization with remarkable amount of silica. The many intersecting volcanic rocks lead one to suggest that the waters are the most radioactive in the vicinity of New York.

